# Migration of aluminum from food contact materials to food—a health risk for consumers? Part II of III: migration of aluminum from drinking bottles and moka pots made of aluminum to beverages

**DOI:** 10.1186/s12302-017-0118-9

**Published:** 2017-04-12

**Authors:** Thorsten Stahl, Sandy Falk, Alice Rohrbeck, Sebastian Georgii, Christin Herzog, Alexander Wiegand, Svenja Hotz, Bruce Boschek, Holger Zorn, Hubertus Brunn

**Affiliations:** 1 Hessian State Laboratory, Am Versuchsfeld 11, 34128 Kassel, Germany; 2 Hessian State Laboratory, Glarusstr. 6, 65203 Wiesbaden, Germany; 3 Institute of Food Chemistry and Food Biotechnology, Justus Liebig University Giessen, Heinrich-Buff-Ring 17, 35392 Giessen, Germany; 4 Institute of Medical Virology, Justus Liebig University, Schubertstraße 81, 35392 Giessen, Germany; 5 Hessian State Laboratory, Schubertstr. 60, 35392 Giessen, Germany

**Keywords:** Aluminum drinking bottles, Aluminum moka pots, Simulants, Coffee preparation, Release limits, Total weekly intake

## Abstract

**Background:**

Drinking bottles and stove-top moka pots made of aluminum have become very popular. Storing drinks in bottles and preparing coffee in a moka pot may result in the migration of aluminum to the beverage.

**Results/Conclusions:**

In a systematic study of aluminum drinking bottles, it has been shown that drinking a mixture of apple juice and mineral water in an aluminum bottle may reach 86.6% of the total weekly intake (TWI) for adults, and drinking tea from an aluminum bottle may exceed the TWI (145%) for a child weighing 15 kg. In contrast, preparing coffee in an aluminum moka pot results in a maximum of 4% to TWI, if an average of 3.17 L coffee is consumed per week, even if the pots are washed in the dishwasher, against the explicit instructions of the manufacturer.

## Background

A list of possible sources of exposure to aluminum can be found in Part I of this report (*Exposure to aluminum, release of aluminum, Tolerable Weekly Intake (TWI), toxicological effects of aluminum*). Also included there are the release or migration limit values [3] for aluminum of 5.00 mg/kg or 5.00 mg/L food or drink, the tolerable weekly intake (TWI) of 1.00 mg aluminum/kg body weight and week [4] as well as the toxicological effects of aluminum. The present Part II deals with the migration of aluminum from drinking bottles and moka pots to beverages. Drinking bottles were tested with tap water, tea made from tea bags, soluble tea drink, and a mixture of apple juice and mineral water. Stove-top moka pots were tested by preparation of coffee as typically performed by consumers and rinsing the pots with water after each use, according to the instructions of the manufacturer. To replicate a worst-case scenario, the moka pots were washed in a household dishwasher after every fifth use, contrary to the recommendation of the manufacturer.

## Methods

A detailed description of sample preparation and analysis can be found in Part I. Therefore, only the experimental details regarding the measurement of aluminum migration from drinking bottles and moka pots to beverages will be presented here.

### Aluminum drinking bottles

Five models of drinking bottles from different manufacturers were tested. Three models were lined with a clear plastic coating and two were not. Three units of each model were tested. The following tests were performed with tap water (pH 7.58) to replicate typical use by consumers. The water was stored at 8 °C in a cold room for 1 week in a 100-L polyethylene (PE) canister with an outlet tap. The canister was rinsed 3 times with 5 L of tap water before use. This water was used to wash and fill the drinking bottles as well as for preparation of tea from tea bags and to dissolve the granulated tea. Three samples of the tap water were stored in 250-mL PE^1^ sample bottles (Heinz Gero Duhme GMBH, Frankfurt, Germany) for blank value testing. Sample bottles were rinsed three times with 100 mL tap water before filling.

#### Tap water

The aluminum drinking bottles were first rinsed with about 200 mL tap water and then filled to the top with tap water from the canister as mentioned above. They were allowed to stand for 24 h at room temperature. An aliquot was then removed and transferred to 250-mL sample bottles and stored at 8 °C until subsequent analysis could be performed.

#### Tea from tea bags

Tea (fruit tea mixture from Meßmer) was prepared twice in a 5-L beaker that had been rinsed with tap water, each time using 3.5 L hot (90 °C) tap water from the canister and 14 tea bags (according to the instructions printed on the package of tea bags: 4 bags per liter water). The tea (pH 3.65) was allowed to steep for 10 min before the bags were removed. The two batches of tea were then poured together and shaken in a 10-L PE canister with outlet tap that had been rinsed three times with 1 L tap water for each rinse. The drinking bottles were then filled to the closure with 40 °C warm tea and the bottles closed with their lids. After a 24-h contact period, aliquots of the tea were transferred to 250-mL sample bottles and stored at 8 °C for subsequent analysis.

#### Granulated tea (instant lemon tea drink)^2^

Two batches of granulated instant lemon tea drink were prepared in a 5-L beaker that had been previously rinsed 3 times with 500 mL tap water. For each batch, one packet (400 g) of granulate was dissolved in 4 L tap water at room temperature. The two batches of the beverage (pH 3.33) were then poured together and shaken for one minute in a 10-L PE canister with outlet tap that had been rinsed three times with 1 L tap water for each rinse. The beverage was then allowed to stand for 12 h at room temperature to assure complete dissolution. The aluminum drinking bottles were filled to the closure with this solution and closed. After a 24-h contact period, aliquots were transferred to 250-mL sample bottles to be used for analysis.

#### Apfelsaftschorle (apple juice with mineral water)^3^

Six 1.5-L bottles of a commercial apple juice and mineral water drink (pH 3.65) were mixed in a 10-L PE canister with outlet tap that had been rinsed three times with 1 L tap water for each rinse and shaken for 1 min. The aluminum drinking bottles were filled to the neck and closed with their lids. After a contact period of 24 h, aliquots (250 mL) of the apple juice and mineral water mixture were transferred to 250-mL sample bottles to be used for subsequent analysis.

### Moka pots

**Fig. 1:**
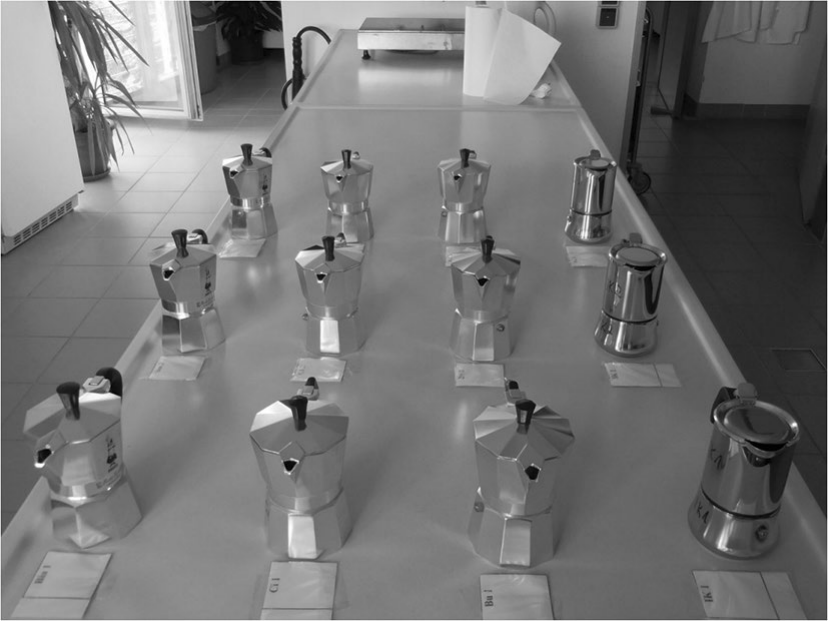
The moka pots tested were made of aluminum or stainless steel (*Stainless steel* The right-hand row)

Four different brands of stove-top moka pots were tested by preparing coffee (three repetitions for each pot) (Fig. 1).

The pots from three manufacturers were made of aluminum and the fourth, for the sake of comparison of potential aluminum migration, was made of stainless steel. Coffee was prepared six times in each pot to determine the difference between a new pot and one that has been used a number of times, on the migration of aluminum.

To emulate the worst-case scenario, the pots were washed in the dishwasher after the fifth preparation of coffee.

Before preparing coffee in the pots, the aluminum content of the coffee itself was determined. To this end, 1.5 kg ground coffee (3 × 0.5 kg packets) was placed in a dry plastic tray that had been rinsed with tap water. The ground coffee was then stirred to homogeneity with a plastic spoon that had also been rinsed with tap water and dried. Care was taken when purchasing the coffee to make certain that all packets had the same batch number. After stirring, three samples of this coffee were removed to be used for determination of aluminum content of the coffee itself. All necessary laboratory equipment was rinsed with tap water before use in order to remove any residual aluminum contamination. Tap water was also used for preparation of the coffee. Thirty liters of tap water were stored in a PE canister with outlet tap that had previously been rinsed with tap water. Coffee was prepared in the aluminum moka pots in exactly the same manner as in the stainless steel pots: 12-g portions of ground coffee were measured out with an accuracy of two decimal places into the strainer basket of the moka pot. The 150 mL water for brewing was drawn from the canister mentioned above through the outlet tap, measured in a graduated cylinder, and poured into the bottom chamber of the coffee maker. Coffee was prepared a total of six times in a row per moka pot. The brewing time was 7 min for all moka pots heated on an electric plate with a temperature of 270 °C. After brewing was complete, the coffee was left in the collecting chamber of the pots for 5 min before transfer to 250-mL sample bottles for subsequent analysis. Allowing the coffee to stand before transfer served to let any particulate coffee to settle in the finished drink. Of the original 150 mL, aliquots of 100 mL were poured through a funnel into the sample bottles. This method assured that no residual coffee particles would find their way into the samples. The remaining coffee in the collection chambers was disposed of and the complete moka pots were rinsed three times each by shaking for 30 s. After the 5th brewing cycle, the moka pots were washed in a standard household dishwasher for 22 min at 50 °C (normal wash cycle with standard household dishwasher detergent tablets). This treatment is specifically not recommended by the manufacturers of the pots and serves as a worst-case condition. Before the pots were reused, they were also rinsed three times each in tap water by shaking for 30 s in order to remove any residue from the dishwasher detergents and to assure that the conditions were the same as for the other brewing cycles.

Evaluation of the moka pot experiments was performed by pairwise comparison of the aluminum concentrations. On the one hand, samples from the individual coffee preparations were compared between brands (independent samples), and on the other hand for each brand the potential differences in aluminum concentration (dependent samples) after the individual coffee preparations were compared. Analysis of possible differences between the brands was performed using the *t* test for independent samples. The *t* test for dependent samples was used to analyze the possible differences between the different coffee preparations from individual brands.

## Results

The aluminum concentrations in the control samples without contact to aluminum-containing equipment (blank values, arithmetic means of *n* = 6) were as follows: in tap water 0.7 µg/L, in tea from teabags 0.72 mg/L, in tea drink made from granulated product 1.74 mg/L, in the apple juice and mineral water mix 0.294 mg/L, and in the ground coffee 5.75 mg/kg. For the sake of clarity, only the blank value-corrected data are shown and were also used for calculations of the aluminum concentration.

### Drinking bottles

**Fig. 2:**
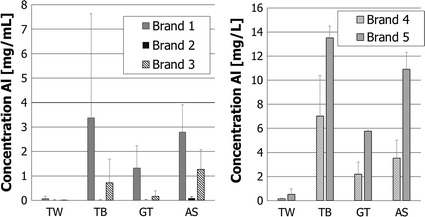
Aluminum concentration in mg/L in the various drinks (*TW* tap water, *TB* tea bag, *GT* granulated tea, *AS* apfelschorle (apple juice mixed with mineral water) after 24-h contact in lined (*left side* brands 1, 2, 3 with *n* = 3) and in unlined drinking bottles (*right side* brands 4 and 5 with *n* = 3)

Three units of each of the bottles from five different manufacturers were tested. The aluminum insides of the bottles from brands 1, 2, and 3 were lined (with a clear plastic coating) and brands 4 and 5 were not lined. The results of the tests are shown in Fig. 2.

It can be seen from Fig. 2 that the unlined bottles (brands 4 and 5) release more aluminum (0.125 mg/L tap water in brand 4, 14.8 mg/L tea (maximum concentration) prepared from bags in brand 5) into the beverages than the lined bottles, brands 1, 2, and 3 (< LOQ tap water in brand 2 and 7.8 mg/L tea (maximum concentration) from bags in brand 1). Based on the arithmetic mean of the results for bottles that were lined and per beverage, the aluminum uptake and percentage to TWI were calculated for a child weighing 15 kg and an adult weighing 70 kg, assuming a daily portion of 500 mL for a period of 1 week (Table 1).

**Table 1:** Aluminum uptake from drinking bottles-child/adult and the respective percentage to TWI

Beverage in aluminum bottle	Mean concentration (mg/L)^a^	Aluminum uptake-child^b^ (mg/week)	Percentage to TWI child	aluminum uptake-adult (mg/week)^c^	Percentage to TWI adult
Lined drinking bottles					
Water (*n* = 9)	0.03	0.12	0.80	0.12	0.17
Tea from bags (*n* = 9)	1.56	5.45	36.3	5.45	7.78
Granulated tea (*n* = 9)	0.44	1.54	10.3	1.54	2.20
Apple juice and mineral water mix (*n* = 9)	1.02	3.58	23.8	3.58	5.11
Unlined drinking bottles					
Water (*n* = 6)	0.30	1.06	7.06	1.06	1.51
Tea from bags (*n* = 6)	6.22	21.8	145	21.8	31.1
Granulated tea (*n* = 6)	2.30	8.05	53.6	8.05	11.5
Apple juice and mineral water mix (*n* = 6)	4.34	15.2	101	15.2	21.7

^a^The results shown here are the arithmetic means of the results from three repetitions of experiments on bottles from all five manufacturers

^b^Data for a child weighing 15 kg consuming a daily portion of 500 mL for a period of 1 week (7 days)

^c^Data for an adult weighing 70 kg consuming a daily portion of 500 mL for a period of 1 week (7 days)

Table 1 shows that an adult will not reach 100% of the TWI with any of the beverages tested in any of the drinking bottles, regardless of whether they are lined or unlined (0.17% for water in a lined bottle, 31.1% for tea made from tea bags in an unlined bottle). A child will reach from 0.8% (water) to 36.3% (tea) of the TWI drinking from a lined bottle. With unlined bottles, a child will exceed the TWI, both with tea from tea bags (145%) as well as with apple juice with mineral water (101%).

### Moka pots

**Fig. 3:**
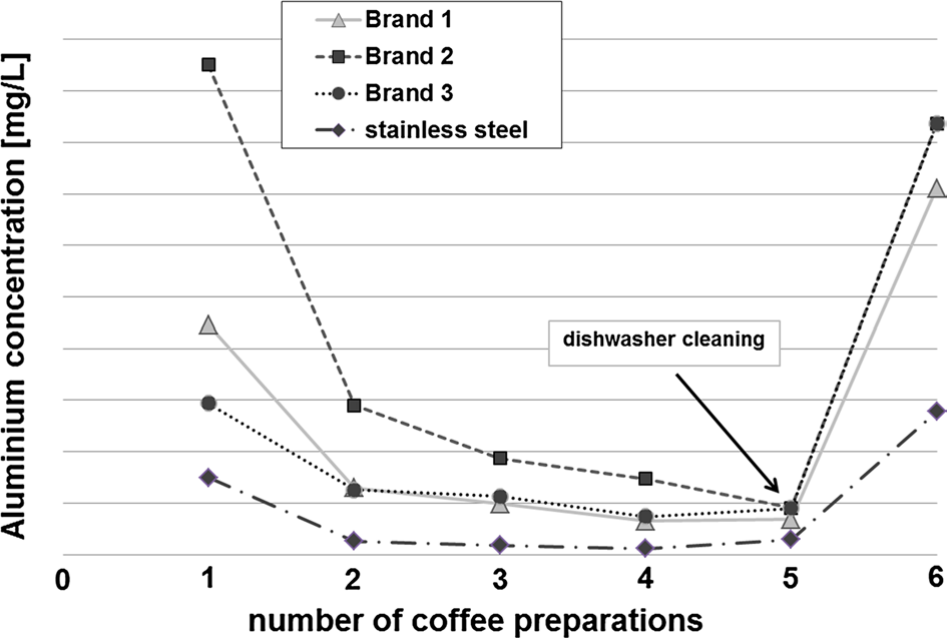
Arithmetic means of aluminum concentration in coffee brewed 6 times in four different brands of moka pots. Pots from brands 1, 2, and 3 were made of aluminum and that from brand 4 was made of stainless steel. After the fifth brew the pots were washed in a dishwasher

Coffee is the favored drink in Germany with 165 L coffee being consumed per person and per year, exceeding the amount of bottled water (140 L) per year. The average German consumes 7.29 kg ground coffee per year (Deutscher Kaffeeverband “[5]” 2014). Brewing coffee in a moka pot is a common and popular method of coffee preparation. According to a market and opinion research poll on the preferred method of coffee preparation in a study from 2014, 4.23 million Germans from age 14 upward most commonly use a moka pot to prepare coffee [6]. Figure 3 shows the arithmetic means of aluminum concentrations in coffee from the moka pots tested. Coffee was brewed six times in each of the four different brands of moka pot. It can be seen from the figure that the mean of aluminum concentrations drops considerably after the first and second brewing. The differences are then minimal between the second and fifth brewing. After the fifth brewing, the pots were washed in a dishwasher, resulting in a considerable increase in aluminum concentration in the sixth brewing.

In order to determine whether there were significant differences in the migration from the different pots, the aluminum concentrations from all six brewings from the pots made of aluminum were compared pairwise with all six brewings from the coffee being made using the stainless steel pots. In all of the pairwise comparisons, it is shown that the arithmetic mean of aluminum concentrations in coffee from the aluminum pots was greater than that in coffee from the stainless steel pots. A statistically significant p value (*p* < 0.05) was found in 13 of the 18 comparisons made. To determine whether the amount of aluminum migrating varied with the increasing numbers of brews, Nos. 1–6 of the individual results for each brewing were combined (=brand independent). Table 2 summarizes the paired comparisons of coffee from the aluminum pots between the numbers of brewings.

**Table 2:** Comparison of aluminum concentration in coffee from aluminum moka pots dependent upon number of times used

Brew number (BN)	BN1–2	BN2–3	BN3–4	BN4–5	BN5–6	BN6–1	BN1–5
Aluminum-moka pot	1 > 2* (*p* = 0.025)	2 > 3 (*p* = 0.055)	3 > 4* (*p* = 0.030)	4 > 5 (*p* = 0.533)	5 < 6* (*p* = 0.000)	6 > 1 (*p* = 0.173)	1 > 5* (*p* = 0.011)

Pair-wise comparisons of coffees from the aluminum moka pots (independent of brand) dependent upon the number of times used to brew (BN)

Significant differences are labeled with an*. The > (<) symbol shows in which of the comparison groups the arithmetic mean was higher (lower). *α* = 0.05. All *n* = 9

A statistically significant difference in aluminum concentration was found in three of the six pairwise comparisons of samples from the serial brewing processes. The difference of the arithmetic mean between the first and second brews was significant. Differences were also observed between the second and third uses; however, these were not statistically significant. The differences between the third and fourth uses were again statistically significant. Concentration decreased consistently until the fifth brewing. The differences were highly significant between Brew Nos. 5 and 6, showing that cleaning in the dishwasher results in significantly higher aluminum concentrations in the coffee. In fact, the concentration was higher after cleaning in the dishwasher than in the brand-new pots (compare Brew Nos. 1 and 6); however, this difference was not statistically significant.

To summarize the results of this series of tests, it must be noted that aluminum migration occurs from aluminum moka pots into the beverage. The arithmetic means of the aluminum concentrations in all pairwise comparisons of coffee from aluminum pots were observed to be higher than those in the coffee from stainless steel pots. This difference was statistically significant (significance level *α* = 0.05) for 13 of 18 pairwise comparisons. The number of times the new aluminum pots were used had a significant influence on the migration: The highest concentration of aluminum was observed the first time the pots were used. From the second to the fifth use, the aluminum concentration in coffee was at a lower level, which can be explained by a passivation of the pot surface. Cleaning the pots in the dishwasher increased the aluminum concentration in the coffee significantly (Brew No. 6). Based on the arithmetic mean of all results per aluminum moka pot (calculation of mean, brand independent) and the stainless steel moka pot for Brew Nos. 1, 3, and 6, the aluminum uptake was calculated for an adult^4^ weighing 70 kg along with the resultant percentage of TWI (Table 3).

**Table 3:** Aluminum uptake and the resultant percentage of TWI

Coffee	Test conditions	Mean concentration (mg/L)	Aluminum uptake adult (mg/week)^b^	Percentage to TWI adult
First brew	Al-pot (*n* = 9)	0.564^a^	1.97	2.81
	Stainless steel pot (*n* = 3)	0.150	0.525	0.750
Third brew	Al-pot (*n* = 9)	0.133^a^	0.469	0.670
	Stainless steel pot (*n* = 3)	0.018	0.063	0.090
Sixth brew	Al-pot (*n* = 9)	0.795^a^	2.79	3.98
	Stainless steel (*n* = 3)	0.278	0.973	1.39

^a^The values shown are the arithmetic means of the results of three repetitions for each of three brands of moka pot

^b^The weekly uptake is based on an adult weighing 70 kg and a daily uptake of 500 mL coffee over a time period of 1 week (7 days)

An average yearly coffee consumption of 165 L, equivalent to 3.17 L per week [5] at the maximum concentration of aluminum (Brew No. 6 after washing the pot in the dishwasher, aluminum concentration 0.795 mg/L coffee) would result in reaching 4% of the TWI.

## Discussion

Studies in a number of European countries (Netherlands, Hungary, Germany, Sweden, and Italy) show that the average aluminum dietary uptake per adult (not including job-related exposure) is between 1.6 and 13 mg per day. This amount corresponds to an exposure of 0.16 to 1.3 mg/kg body weight per week for an adult weighing 70 kg [1, 4] which is equivalent to 16–130% of the TWI. It must be noted, however, that there are large differences in the average contamination between individual countries, and it is not always evident in the various studies whether or not drinking water was included in the calculations. A large variation in individual exposure certainly can be expected as a result of differences in environment, soil contamination, dietary habits, or the consumption of foods with additives that contain aluminum [1, 4]. Children consume more nourishment per body weight than adults and therefore represent the group with the highest potential for exposure to aluminum per kilogram body weight [1, 4]. Studies in France show that the estimated exposure for children between 3 and 15 years amounts to 0.7 mg/kg BW/week (corresponding to 70% of the TWI), and for infants and toddlers between 1.5 and 4.5 years the exposure is 23 mg/kg BW/week (corresponding to 230% TWI). In the UK, studies show that the value is 1.7 mg/kg BW/week for children in the age group of 4–18 years (corresponding to 170% TWI).

In Health Evaluation No. 033/2007 [2], the German Federal Institute for Risk Assessment (BfR) clearly states “No danger of contracting Alzheimer’s disease from aluminum in household utensils.” Furthermore, this document states that there is no scientific evidence indicating a connection between aluminum uptake from foodstuffs, including drinking water, pharmaceuticals, or cosmetics, and Alzheimer’s disease. No increases in the frequency of amyloid plaques in the brain have been found in dialysis patients or in aluminum workers, both groups of people with extensive contact with aluminum. The BfR, therefore, does not recognize a health danger for consumers through aluminum uptake from food and cooking utensils or cosmetics [2]. The BfR does recommend that consumers avoid the use of aluminum pots or dishes for acidic or salted foodstuffs such as apple sauce, rhubarb, tomato puree, or salt herring due to the increased solubility of aluminum under the influence of acids and salts, thus prophylactically avoiding the “unnecessary ingestion” of aluminum [2].

In the present study, two household utensils, drinking bottles and moka pots made of aluminum, were examined in regard to the migration of aluminum to food or drink. To summarize the results presented here, it can be said that human exposure with aluminum resulting from properly used drinking bottles is negligible. Acidic beverages such as apple juice with mineral water or tea should not be used in aluminum drinking bottles. This is particularly important for children. The tests here show that the TWI will be (145%) exceeded for a child weighing 15 kg drinking 500 mL brewed tea from an unlined aluminum bottle. Migration of aluminum from lined bottles is much less. The specific release limit (SRL) of 5.00 mg/kg or 5.00 mg/L was not exceeded by any of the lined bottles tested. Unlined bottles, in contrast, did exceed these limits when filled with acidic beverages. Additional human inner aluminum exposure through the proper use of aluminum moka pots is negligible. Even under the worst-case scenario of washing the moka pots in a dishwasher, the uptake amounts to only 4% of TWI. The manufacturers expressly warn not to clean the aluminum moka pots in the dishwasher.

## Footnotes

1 In the following, the term “250-mL sample bottles” will be used since all bottles are made of polyethylene.

2 List of ingredients according to the manufacturer: sugar, glucose, acidifier citric acid, black tea extract cold-water soluble 1.28%, vitamin C, lemon extract powder (maltodextrin, lemon juice concentrate), aroma.

3 List of ingredients according to the manufacturer: apple juice from apple juice concentrate, carbonated drinking water, and 60% fruit concentration.

4 The percentage to TWI was not calculated for a child weighing 15 kg under the assumption that children do not drink coffee.
